# Comparison of engineered *Escherichia coli* AF1000 and BL21 strains for *(R)-*3-hydroxybutyrate production in fed-batch cultivation

**DOI:** 10.1007/s00253-019-09876-y

**Published:** 2019-05-18

**Authors:** Mariel Perez-Zabaleta, Mónica Guevara-Martínez, Martin Gustavsson, Jorge Quillaguamán, Gen Larsson, Antonius J. A. van Maris

**Affiliations:** 1 School of Engineering Sciences in Chemistry, Biotechnology, and Health (CBH), Department of Industrial Biotechnology, KTH Royal Institute of Technology, AlbaNova University Center, SE-10691 Stockholm, Sweden; 2 Center of Biotechnology, Faculty of Science and Technology, Universidad Mayor de San Simón, Cochabamba, Bolivia

**Keywords:** *Escherichia coli*, (*R*)-3-hydroxybutyrate, Acetate, Nitrogen limitation, Fed batch, BL21

## Abstract

Accumulation of acetate is a limiting factor in recombinant production of (*R*)-3-hydroxybutyrate (3HB) by *Escherichia coli* in high-cell-density processes. To alleviate this limitation, this study investigated two approaches: (i) deletion of phosphotransacetylase (*pta*), pyruvate oxidase (*poxB*), and/or the isocitrate lyase regulator (*iclR*), known to decrease acetate formation, on bioreactor cultivations designed to achieve high 3HB concentrations. (ii) Screening of different *E. coli* strain backgrounds (B, BL21, W, BW25113, MG1655, W3110, and AF1000) for their potential as low acetate-forming, 3HB-producing platforms. Deletion of *pta* and *pta-poxB* in the AF1000 strain background was to some extent successful in decreasing acetate formation, but also dramatically increased excretion of pyruvate and did not result in increased 3HB production in high-cell-density fed-batch cultivations. Screening of the different *E. coli* strains confirmed BL21 as a low acetate-forming background. Despite low 3HB titers in low-cell-density screening, 3HB-producing BL21 produced five times less acetic acid per mole of 3HB, which translated into a 2.3-fold increase in the final 3HB titer and a 3-fold higher volumetric 3HB productivity over 3HB-producing AF1000 strains in nitrogen-limited fed-batch cultivations. Consequently, the BL21 strain achieved the hitherto highest described volumetric productivity of 3HB (1.52 g L^−1^ h^−1^) and the highest 3HB concentration (16.3 g L^−1^) achieved by recombinant *E. coli*. Screening solely for 3HB titers in low-cell-density batch cultivations would not have identified the potential of this strain, reaffirming the importance of screening with the final production conditions in mind.

**Electronic supplementary material:**

The online version of this article (10.1007/s00253-019-09876-y) contains supplementary material, which is available to authorized users.

## Introduction

**Fig. 1:**
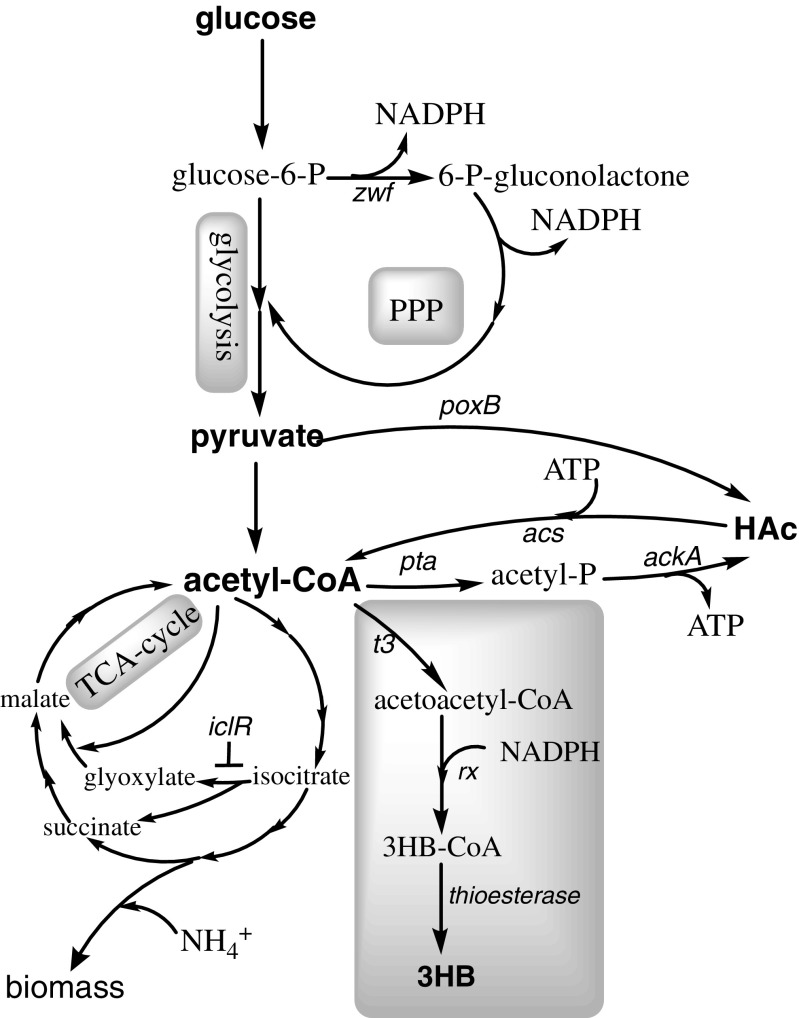
Simplified metabolic scheme showing pathways of (*R*)-3-hydroxybutyrate (3HB), acetic acid (HAc), and biomass formation for *E. coli* under aerobic conditions. Genes notation refer to glucose-6-phosphate dehydrogenase (*zwf*), pyruvate oxidase (*poxB*), phosphotransacetylase (*pta*), acetate kinase (*ackA*), acetyl-CoA synthase (*acs*), isocitrate lyase regulator (*iclR*), β-ketothiolase from *H. boliviensis* (*t3*), and acetoacetyl-CoA reductase from *H. boliviensis* (*rx*), native *E. coli* acyl-CoA thioesterase (thioesterase). Abbreviations refer to (*R*)-3-hydroxybutyrate (3HB), acetic acid (HAc) and pentose phosphate pathway (PPP)

(*R*)-3-hydroxybutyrate (3HB) is the monomer of the well-known bioplastic poly-3-hydroxybutyrate (PHB) and has additionally gained considerable attention for its potential as an intermediate in the synthesis of chiral chemicals (Kashiwaya et al. 2000; Suzuki et al. 2001; Tseng et al. 2009). 3HB can be produced by chemical synthesis, de-polymerization of PHB, or by recombinant microorganisms (de Roo et al. 2002; Jaipuri et al. 2004; Lee and Lee 2003). Among these methods, recombinant production is the most desired because it is a one-step process and avoids the use of non-environmentally friendly chemicals. The pathway to produce recombinant 3HB from acetyl-CoA contains three enzymes: (i) β-ketothiolase that catalyzes the condensation of two molecules of acetyl-CoA to acetoacetyl-CoA, (ii) acetoacetyl-CoA reductase that catalyzes the reduction of acetoacetyl-CoA to 3HB-CoA, and (iii) acyl-CoA thioesterase that releases CoA from 3HB-CoA to form 3HB (Fig. 1).

*Escherichia coli* is an interesting production platform for 3HB, due to being a robust microorganism with simple growth requirements, good genetic accessibility, and the presence of native thioesterases required to hydrolyze 3HB-CoA (McMahon and Prather 2014; Tseng et al. 2009). Heterologous expression of β-ketothiolase (*t3*) and acetoacetyl-CoA reductase (*rx*) from *Halomonas boliviensis*, which is an efficient PHB producer (1.1 g L^−1^ h^−1^ PHB) (Quillaguamán et al. 2008), has previously led to high 3HB production by engineered *E. coli* AF1000 (Guevara-Martínez et al. 2015; Jarmander et al. 2015; Perez-Zabaleta et al. 2016). An important factor limiting recombinant production of 3HB by *E. coli* has been the formation of acetate (Gao et al. 2002; Liu et al. 2007; Perez-Zabaleta et al. 2016; Tseng et al. 2009). Production of acetate not only competes with product formation for the substrate, thereby decreasing 3HB yield, but also lowers the growth rate even at concentrations as low as 0.5 g L^−1^ (Nakano et al. 1997), making it difficult to obtain high-cell-density cultures and to scale-up the processes.

One approach to limit acetate formation is to adjust bioprocess conditions and/or medium composition (De Mey et al. 2007). However, although application of glucose limitation dramatically reduces acetate formation (Shiloach and Fass 2005), it also decreases glycolytic flux and acetyl-CoA supply and thereby also 3HB formation. In another study, competition between growth and product formation was modulated by nitrogen limitation in fed batch while maintaining high glucose concentrations (Guevara-Martínez et al. 2015). However, while resulting in decreased growth and improved 3HB titers, this also resulted in increased acetate concentrations (Guevara-Martínez et al. 2015). Although partially successful, these examples illustrate how avoiding acetate formation through process adjustments alone tends to go at the expense of production capacity (De Mey et al. 2007), indicating that an *E. coli* strain with an inherent lower tendency to produce acetate is attractive for 3HB production.

Metabolic engineering is another approach to decrease by-product formation, and genetic approaches for decreasing acetate formation have been extensively reviewed (De Mey et al. 2007). Previously described strategies that decrease acetate formation, by engineering the PTS system for decreased glucose uptake rates (Bäcklund et al. 2008; De Anda et al. 2006), or by heterologous expression of hemoglobin (Pablos et al. 2014), are expected to negatively affect the availability of pyruvate and/or acetyl-CoA. Downstream of the pyruvate/acetyl-CoA node, the two main pathways for acetate formation in *E. coli* under aerobic conditions are pyruvate oxidase (*poxB*) and phosphotransacetylase (*pta*)-acetate kinase (*ackA*) (Fig. 1). To a lesser extent, acetate formation can also be attributed to other acetate-producing pathways such as *N*-acetylornithine deacetylase (Javid-Majd and Blanchard 2000) and citrate lyase (Kakuda et al. 1994). Deletion of *pta* and *poxB* has previously been shown to positively affect PHB production in *E. coli* (Chang et al. 1999; Jian et al. 2010). In addition to these two commonly accepted methods, there is also diversity in the level of acetate formation between different *E. coli* strain backgrounds. For instance, strains BL21 and B are known to produce lower amounts of acetate, which has been attributed to a lower gene expression of pyruvate oxidase (*poxB*) and the isocitrate lyase regulator (*iclR*) compared to K-12 strains (Daegelen et al. 2009; Phue et al. 2005; van de Walle and Shiloach 1998; Waegeman et al. 2011).

This study aims to investigate the specific impact of metabolic engineering approaches as well as strain diversity for decreased acetate formation on 3HB production in high-cell-density fed-batch processes. In the first part of this study, the impact of deletion of *poxB*, *pta*, and/or *iclR* on high-density 3HB-producing fed-batch cultivations was investigated in a previously constructed *E. coli* AF1000 strain background containing *H. boliviensis* β-ketothiolase (*t3*) and acetoacetyl-CoA reductase (*rx*) (Jarmander et al. 2015), as well as overexpressed glucose-6-phosphate dehydrogenase (*zwf*) to enhance the NADPH supply (Perez-Zabaleta et al. 2016). In the second part of this study, seven different *E. coli* strain backgrounds (B, BL21, W, MG1655, W3110, BW25113, and AF1000) were first investigated to find the most promising 3HB production hosts. After initial screening, the most promising strains were transformed with the 3HB-pathway plasmids and tested in high-cell-density cultivation to evaluate 3HB titers and productivity.

## Materials and methods

### Strains and plasmids

*Escherichia coli* strains investigated in this study are shown in Table 1. Working cell banks were prepared with cells in their exponential growth phase and were kept at − 80 °C in minimal medium with 25% (*v*/*v*) glycerol. Plasmids are also listed in Table 1 and were maintained at − 20 °C. The plasmid for 3HB production was pJBGT3RX (Jarmander et al. 2015), which carried β-ketothiolase gene (*t3*; WP_007111820) and acetoacetyl-CoA reductase gene (*rx*; WP_007111780) from *H. boliviensis*. Plasmid pBADzwf was used for enhancing NADPH supply, which contains the overexpression of glucose-6-phosphate dehydrogenase encoded by the gene *zwf* (Perez-Zabaleta et al. 2016). Plasmids were transformed into *E. coli* electrocompetent cells by electroporation at 1.8 kV in pre-cooled cuvettes with 0.1 cm gap (Bio-Rad, Hercules, CA). Fifty microliters of cells was mixed with 100 ng of plasmid purified with GeneJET Plasmid Miniprep Kit (Thermo Fisher Scientific, Loughborough, UK). The electroporated cells were recovered in 950 μL of Luria Bertani medium (Miller 1972) and after incubating for 1 h, they were plated on LB-agar (LB with 1.5% agar) with the respective antibiotic. One single colony was used to make master and working cell banks.

**Table 1:** List of *E. coli* strains and plasmids used in this study

Strains/plasmids	Genotype	Source of reference(s)
*E. coli* strains		
B	B	ATCC # 11303
BL21	B, *F*^*−*^*ompT hsdS(rb*^*–*^*mb*^*–*^*)gal dcm*	SIGMA # 27-1542-01
MG1655	K-12, *F*^*–*^*λ*^*–*^*ilvG*^*–*^*rfb-50 rph-1*	CGSC # 6300
BW25113	K-12*,* Δ*(araD-araB)567* Δ*(rhaD-rhaB)568* Δ*lacZ4787 (:rrnB-3) hsdR514 rph-1*	CGSC # 7636
W	W	ATCC # 9637
AF1000	K-12, MC4100 *relA*^*+*^	(Sanden et al. 2003)
W3110	K-12, *F*^*–*^*λ*^*–*^*mcrA mcrB IN(rrnD-rrnE)1*	ATCC # 27325
AF1000Δ*iclR*	AF1000 with deletion of isocitrate lyase regulator (*iclR*)	This study
AF1000Δ*pta*	AF1000 with deletion of phosphotransacetylase (*pta*)	This study
AF1000Δ*poxB*	AF1000 with deletion of pyruvate oxidase (*poxB*)	This study
AF1000Δ*pta*Δ*poxB*	AF1000 with deletion of phosphotransacetylase (*pta*) and pyruvate oxidase (*poxB*)	This study
BL21Δ*pta*	BL21 with deletion of phosphotransacetylase (*pta*)	This study
BL21Δ*poxB*	BL21 with deletion of pyruvate oxidase (*poxB*)	This study
BL21Δ*pta*Δ*poxB*	BL21 with deletion of phosphotransacetylase (*pta*) and pyruvate oxidase (*poxB*)	This study
Plasmids		
pJBGT3RX	pACYC184 derivative, *ori* p15A, lacUV5 promoter, lacIq repressor, Cm^R^	(Jarmander et al. 2015)
pBADzwf	pBAD plasmid, *ori* pBR322, araBAD promoter, Amp^R^	(Perez-Zabaleta et al. 2016)
pKD3	FRT-Cm^R^-FRT, oriR6K, Amp^R^	Addgene # 45604, (Datsenko and Wanner 2000)
pKD4	FRT-Kan^R^-FRT, oriR6K, Amp^R^	Addgene # 45605, (Datsenko and Wanner 2000)
pSIJ8	pKD46 derivative, temperature sensitive, arabinose inducible λ Red recombinase, rhaRS-prha-FLP, Amp^R^	Addgene # 68122, (Jensen et al. 2015)

### Gene deletions

Gene knockouts were performed in *E. coli* AF1000 and BL21. The plasmids used for gene disruption were (i) pSIJ8, which contains the lambda red recombineering genes and flippase recombinase (FLP) (Jensen et al. 2015), (ii) pKD3, template for the FRT-flanked cat cassette, and (iii) pKD4, template for the FRT-flanked kan cassette (Datsenko and Wanner 2000) (Table 1). Gene knockout primers were designed with 50-nt homologous to regions adjacent to the target genes (*pta*, *poxB*, and *iclR*) and around 20-nt priming sequences for pKD3 or pKD4 (Table S1, online resource). Primers were synthetized by IDT (Leuven, Belgium). In order to generate the deletions, the helper plasmid pSIJ8 was inserted in both strains, AF1000 and BL21. All following knockout steps were performed at 30 °C in order to maintain the temperature-sensitive pSIJ8, and performed according to the method described by Jensen et al. 2015. LB medium was used to recover the electroporated cells. Once the deletion was confirmed by PCR, the knockout strains were cured of the helper plasmid by cultivation at 37 °C. DNA sequencing was performed to confirm the deletions. Sequencing primers are listed in Table S1 (online resource).

### Cultivation medium

With the exception of nitrogen-depleted batch cultivations, all cultivations were started on minimal salts media containing 7 g L^−1^ (NH_4_)_2_SO_4_ (Merck, Darmstadt, Germany), 1.6 g L^−1^ KH_2_PO_4_ (VWR International, Leuven, Belgium), 6.6 g L^−1^ Na_2_HPO_4_·2H_2_O (VWR International), and 0.5 g L^−1^ (NH_4_)_2_-H-(citrate) (Merck). Glucose (Thermo Fisher Scientific, Waltham, MA) was used as the sole carbon source and, at the start of all bioreactor experiments, 15 g L^−1^ of glucose was added to the minimal medium from a heat sterilized stock solution of 500 g L^−1^. During nitrogen limitation or nitrogen depletion, additional sugar was added as indicated below. The sterile minimal medium was supplemented with 1 ml L^−1^ of trace elements and 1 ml L^−1^ of 1 M MgSO_4_·7H_2_O (Merck), both filter-sterilized (0.2 μm, VWR collection) before addition. Composition of the trace elements solution was 0.5 g L^−1^ CaCl_2_·2H_2_O (Merck), 16.7 g L^−1^ FeCl_3_·6H_2_O (Merck), 0.18 g L^−1^ ZnSO_4_·7H_2_O (Merck), 0.16 g L^−1^ CuSO_4_·5H_2_O (Merck), 0.11 g L^−1^ MnSO_4_·H_2_O (Merck), 0.18 g L^−1^ CoCl_2_·6H_2_O (Merck), and 20.1 g L^−1^ Na_2_-EDTA (Merck). Antifoam B125 (BASF, Stockholm, Sweden) was added to the growth medium as required during cultivation.

For *E. coli* cells harboring plasmid pJBGT3RX, 50 μg ml^−1^ chloramphenicol (Sigma-Aldrich, St Louis, MO) was added to the cultivation medium and 3HB production was induced with 200 μM isopropyl β-D-1-thiogalactopyranoside (IPTG) (VWR International). For plasmid pBADzwf, 100 μg ml^−1^ ampicillin (Sigma-Aldrich) was added to the growth medium and induction was performed with 2 mg L^−1^ L-arabinose (Sigma-Aldrich). For nitrogen-depleted batch cultivations, the following adjustments were made to the medium: 0.5 g L^−1^ (NH_4_)_2_-H-(citrate) was replaced by 0.7 g L^−1^ (Na)_3_-H-(citrate)·2H_2_O (Merck) and the initial (NH_4_)_2_SO_4_ concentration was reduced to 3 g L^−1^ for wild-type strains and 2 g L^−1^ for knockout strains.

### Inoculation and growth conditions

For cell propagation, working cell stocks stored at − 80 °C were first activated by growing them in overnight in minimal medium with 5 g L^−1^ glucose with the respective antibiotics in baffled shake flasks at 37 °C in an orbital shaker (Infors, Basel, Switzerland) at 180 rpm until a final optical density at 600 nm (OD_600_) of 2 was reached. After 16 h of cultivation, the inoculum was added to stirred tank bioreactors at 5% (*v*/*v*) (5 ml inoculum per 100 ml cultivation broth) to attain an initial OD_600_ of 0.1. Bioreactor experiments were performed in stainless-steel stirred tank bioreactors of 1 L (GRETA-system, Belach Bioteknik, Skogås, Sweden) or 15 L (Belach Bioteknik), as indicated below. The cultivations were induced for 3HB production and *zwf* overexpression after one generation (OD_600_ = 0.2) and this point was considered the time zero of the experiments. The cultivation temperature was 37 °C, and the pH was kept at 7 by automatic titration with 5 M NaOH (Merck) solution. Cultivations were performed aerobically and the dissolved oxygen was maintained above 20% by manually increasing the airflow and stirring speed when needed. All bioreactor cultivation experiments were performed in duplicate unless stated otherwise. Samples of 17 ml were collected each hour.

### Batch experiments

Batch experiments were performed in parallel stainless-steel bioreactors of 1 L (GRETA-system, Belach Bioteknik, Skogås, Sweden) with a working volume of 800 ml. All batch experiments were performed in standard medium with an initial glucose concentration of 15 g L^−1^. In the batch experiments ending with a nitrogen-depleted 3HB production phase, another 15 g L^−1^ glucose was added upon nitrogen-depletion.

### Nitrogen-limited fed batch

These experiments were performed in a 15-L stirred tank stainless-steel bioreactor (Belach Bioteknik) with an initial working volume of 8 L. Cultivations consisted of a batch phase with repeated manual addition of glucose and (NH_4_)_2_SO_4_, henceforth referred to as repeated batch, followed by a nitrogen-limited growth phase (N-limited). Upon reaching an OD_600_ of 20, an extra 5 g L^−1^ (NH_4_)_2_SO_4_ was added and at an OD_600_ of 30 an extra 1.2 g L^−1^ (NH_4_)_2_SO_4_ was added. In total, 13.2 g L^−1^ (NH_4_)_2_SO_4_ was used in the repeated batch phase. Starting with an initial 15 g L^−1^ of glucose, glucose was monitored hourly by test strips (Siemens, Bayer Uristix, Ref 2857) to maintain a concentration > 10 g L^−1^ by manual addition of a 500 g L^−1^ glucose stock solution. The total amount of glucose added during the repeated batch phase depended on the metabolic activity and was 75 g L^−1^ for AF1000-T3Rxzwf, 55 g L^−1^ for AF1000Δ*pta*-T3Rxzwf, 60 g L^−1^ for AF1000*Δpta*Δ*poxB*-T3Rxzwf, and 60 g L^−1^ for BL21-T3Rxzwf. When the nitrogen was depleted at the end of the repeated batch phase, as indicated by an increase in DOT, the nitrogen-limited fed-batch phase was started. At this point, the flow rate of the feed (F; kg h^−1^) was calculated by Eq. (1.1):

1.1 $$ F=\frac{\mu^{\ast }{x}^{\ast }V}{S_N^{\ast }{Y}_{x/N}} $$

where *μ* (h^−1^) is the specific growth rate calculated for the last three sample points before feed start, *x* (g L^−1^) is the cell concentration at feed start as estimated by OD_600_, *V* (L) is the reactor volume at feed start, *S*_N_ (g kg^−1^) is the concentration of (NH_4_)_2_SO_4_ in the feed solution, and *Y*_*x*/N_ (g g^−1^) is the yield of cells per (NH_4_)_2_SO_4_ consumed as calculated from previous experiments. The feed was kept constant and its composition was 394 g kg^−1^ glucose and 130 g kg^−1^ (NH_4_)_2_SO_4_. Upon each increase of 10 OD_600_, 1 ml L^−1^ of trace elements solution and 1 ml L^−1^ of 1 M MgSO_4_ were added manually. In the specific case of BL21-T3Rxzwf, an extra 40 g L^−1^ of glucose was manually added during the nitrogen-limited phase.

### Analysis of cultivation samples

The OD_600_ was monitored in a spectrophotometer (Genesys 20, Thermo scientific) after diluting the cultivation broth with saline solution, 0.9%, *w*/*v* NaCl (Scharlau, Barcelona, Spain) to an approximate OD_600_ of 0.1. Cell dry weight (CDW) was determined in triplicate by taking 5 ml samples into pre-weighed, dried glass tubes, which were centrifuged at 4500 rpm in a tabletop centrifuge (Hermle Z 206A, Wehingen, Germany) for 10 min. The supernatant was decanted, filtered (0.2 μm, VWR collection), and stored at − 20 °C until further analysis. The resulting cell pellets were dried overnight at 105 °C, allowed to cool at room temperature in a desiccator, and were subsequently weighed. Supernatant concentrations of glucose, pyruvate, 3HB, acetate, and other possible by-products such as citrate, lactate, succinate, formate, malate, and fumarate were measured by high-performance liquid chromatography (HPLC) (Alliance Waters 2695, Stockholm, Sweden) using column HPX-87H (Bio-Rad, Hercules, CA). The glucose was measured using a refractive index detector (Waters 2414) at 410 nm and organic acids with a photodiode array detector (Waters 2996) at 210 nm. Twenty microliters of injection samples was analyzed with the following operating conditions of the HPLC: 0.5 ml min^−1^ flow rate, 0.008 N H_2_SO_4_ (Sigma-Aldrich) mobile phase, 30 min running time and the column was at room temperature. Ammonium concentrations were measured using an enzymatic ammonia assay kit (Cat No. K-AMIAR, Megazyme, Leinster, Ireland) according to the manufacturers’ protocol.

### Calculation of rates

To determine the specific growth rate (μ), the natural logarithm of OD_600_ was plotted as a function of time and μ was the resulting slope. Equation (1.2) was used to calculate the yields (*Y*_p/s_), where p was the amount of products, either acetic acid (HAc) or 3HB and s was the amount of glucose consumed. Where required, total amounts were corrected for volume changes due to media feed and base addition. Production rates were fitted in a first- or second-order functions by least-squares regression; thus, the grams of product of each sample was plotted versus time and Eq. (1.3) was obtained. The derivative of function (1.3) was divided by the cultivation volume (*V*_t_) at each sample point, which resulted in Eq. (1.4) and it was used to calculate the volumetric productivity rate of HAc and 3HB (*r*_p_). The specific production rate (*q*_p_) was calculated using Eq. (1.5), where *x*_t_ (g L^−1^) is the cell concentration at each sample point and *r*_p_ (g L^−1^ h^−1^) is the volumetric production rate obtained with Eq. (1.4).

1.2 $$ {y}_{p/s}=\frac{dp}{ds} $$

1.3 $$ p=f(t) $$

1.4 $$ {r}_p=\frac{f^{\hbox{'}}(t)}{V_t} $$

1.5 $$ {q}_p=\frac{r_p}{x_t}=\frac{f^{\hbox{'}}(t)}{x_t\ast {V}_t} $$

## Results

### Impact of deletion of *pta*, *poxB*, and/or *iclR* on 3HB production in high-cell-density nitrogen-limited fed-batch cultivation

**Fig. 2:**
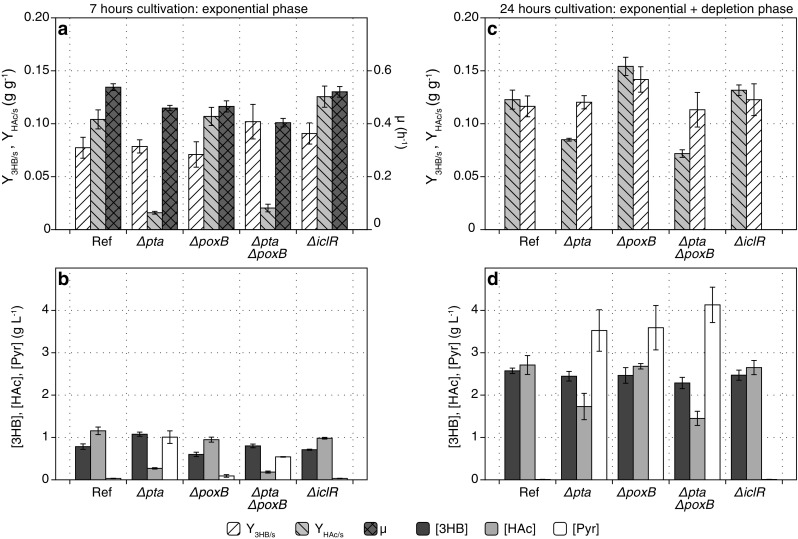
Evaluation of (*R*)-3-hydroxybutyrate and acetate formation of *pta*, *poxB*, and/or *iclR* deletions in the AF1000 strain background. Experiments were performed in batch bioreactor cultivations designed with two phases, exponential growth phase and nitrogen-depleted phase. Both plasmids, pJBGT3RX and pBADzwf, were inserted in the AF1000 reference and the knockout strains. Boxes (a) and (b) show cultivations parameters during the exponential growth phase. Boxes (c) and (d) show the parameters after 24 h of cultivation. Boxes (a) and (c) show the yield of acetate on glucose (Y_HAc/s_) and the yield of (*R*)-3-hydroxybutyrate on glucose (Y_3HB/s_). Additionally, box (a) shows the specific growth rate (μ). Boxes (b) and (d) show the concentrations of (*R*)-3-hydroxybutyrate ([3HB]), acetate ([HAc]) and pyruvate ([Pyr]). Bars represent the average and mean deviation of duplicate cultivations

To investigate the impact of the deletions of enzymes involved in aerobic acetate formation during 3HB production, *pta*, *poxB*, and/or *iclR* were deleted in the AF1000 strain background, which is derived from *E. coli* MC4100 (Sanden et al. 2003). The variants contained both the 3HB-producing enzymes (pJBGT3RX), as well as the overexpressed NADP-dependent glucose-6-phosphate dehydrogenase (pBADzwf). As a first screening, the strains were tested for 3HB and acetate formation during exponential growth as well as during nitrogen-depletion (Fig. 2). The first 7 h of the experiments covered most of the exponential growth phase of the cultivations (Fig. 2a, b), whereas the samples at 24 h covered both phases (Fig. 2c, d). Deletion of *pta* or *poxB* slightly decreased the specific growth rate, while a double deletion in *pta* and *poxB* decreased the specific growth rate to 0.40 h^−1^ (Fig. 2a). During exponential growth, deletion of *pta* decreased the acetate concentration by 77%, while deletion of *poxB* only resulted in a minor reduction of 18% (Fig. 2a). Combined deletion of *pta* and *poxB* resulted in a slight decrease in the acetate concentration by 85% compared to deletion of *pta* alone (Fig. 2b). This indicates that, in line with literature (Dittrich et al. 2005), the *ackA-pta* pathways is the dominant acetate-forming pathway under these conditions. However, in contrast to the reference and Δ*iclR* strain, variants with *pta* or *pta*-*poxB* deletions started to accumulate pyruvate in the exponential phase (Fig. 2b). During the nitrogen-depleted phase, the deletion strains produced similar amounts of acetate compared to the reference strain, resulting in 36% and 47% differences in the final acetate concentration for Δ*pta* and Δ*pta*Δ*poxB*, respectively, while no significant changes in 3HB were observed over the entire 24-h cultivation (Fig. 2c, d). Additionally, during the nitrogen-depleted phase, the pyruvate production in Δ*pta*, Δ*poxB*, and Δ*pta*Δ*poxB* strains actually exceeded acetate production in the reference strain (Fig. 2d). On the other hand, the physiology of the Δ*iclR* strain and the reference strain did not differ significantly in any of the two cultivation phases.

**Fig. 3:**
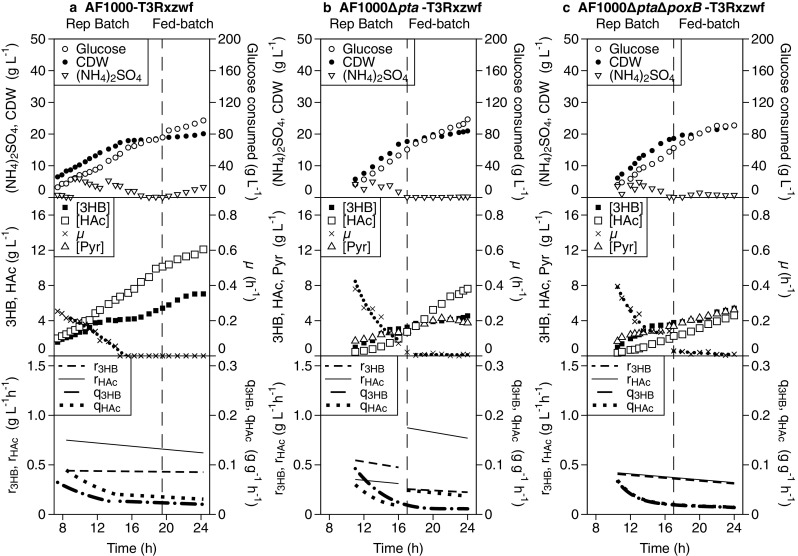
Nitrogen-limited fed-batch cultivations to evaluate (*R*)-3-hydroxybutyrate and acetate formation by (a) AF1000 (b) AF1000Δ*pta* and (c) AF1000Δ*pta*Δ*poxB.* Experiments were performed in fed-batch bioreactor cultivations with constant feed and were designed with a repeated batch phase followed by a nitrogen-limited phase. The vertical dashed line marks the shift between repeated batch and nitrogen-limited fed batch. Samples were taken from OD_600_ = 10. The strains were transformed with both plasmids, pJBGT3RX and pBADzwf. Symbols refer to cell dry weight (CDW, filled circles), accumulative glucose consumed (Glucose, open circles), (NH_4_)_2_SO_4_ concentration ((NH_4_)_2_SO_4_, inverted open triangles), specific growth rate (μ, crosses and dotted line), (*R*)-3-hydroxybutyrate concentration ([3HB], closed squares), acetate concentration ([HAc], open squares), pyruvate concentration ([Pyr], open triangles). The specific 3HB production rate (q_3HB_, dash-dotted line), volumetric 3HB productivity (r_3HB_, dashed line), specific acetate production rate (q_HAc_, dotted line), and volumetric acetate productivity (r_HAc_, solid line) as calculated from spline-fit of the raw data. Experiments were performed in duplicate; one representative experiment is shown in this figure and the duplicate is shown in Fig. S1 (online resource)

Although the impact of the deletions on the 3HB yield in batch was underwhelming, the observed shift from acetate to pyruvate formation, in combination with the lower pK_a_ (2.5 versus 4.76) and oleyl-water partitioning coefficient (0.12 versus 0.22) of pyruvate compared to acetate (Collander 1951; Dawson 1959), might decrease weak-acid toxicity at the later stages in high-cell-density nitrogen-limited fed-batch cultures. For this reason, AF1000 strains containing pJBGT3RX and pBADzwf and knockouts in *pta* and *pta*-*poxB* were compared to the reference strain in a process consisting of a batch phase with repeated manual addition of glucose and (NH_4_)_2_SO_4_, below referred to as repeated batch, which allows rapid biomass formation at maximum specific growth rate, followed by a nitrogen-limited fed-batch phase to improve 3HB production (Fig. 3). In this process, the growth of AF1000-T3Rxzwf quickly deteriorated due to acetate accumulation and the growth rate was practically zero when the acetate concentration reached 6.74 g L^−1^ after 14.6 h, and when the repeated batch phase ended the acetate concentration had increased even further to 10.14 g L^−1^ (Fig. 3a). This inhibition of growth also caused the accumulation of (NH_4_)_2_SO_4_ during the feed phase (Fig. 3a). Compared to the reference strain, the acetate concentration at the end of the repeated batch phase was decreased by 73% (2.73 g L^−1^) for Δ*pta* and by 78% for Δ*pta*Δ*poxB* strain (2.27 g L^−1^) (Fig. 3b, c). The slower increase of the acetate concentration for the Δ*pta* and Δ*pta*Δ*poxB* strains during the repeated batch phase resulted in higher specific growth rates and a slightly shorter duration of this phase (Fig. 3b, c) compared to the reference strain (Fig. 3a). The pyruvate concentrations of the reference, Δ*pta*, and Δ*pta*Δ*poxB* strains were, respectively, below the detection limit, 2.84 g L^−1^ and 3.37 g L^−1^.

During the nitrogen-limited fed-batch phase, the CDW of all strains increased only slightly (Fig. 3), which together with the volume increase resulted in a slight decrease of the volumetric productivities (r_HAc_ and r_3HB_) with time. Upon completion of the fed-batch phase after a total cultivation time of 24 h, acetate formation was reduced by 37% and 62%, respectively, in the Δ*pta* and Δ*pta*Δ*poxB* strains compared to the AF1000 reference strain (Fig. 3). However, as seen in the batch phase, pyruvate production was dramatically increased for both Δ*pta* and Δ*pta*Δ*poxB* in the fed-batch phase, while the 3HB titers and production rates of Δ*pta* and Δ*pta*Δ*poxB* were not improved compared to the reference strain (Fig. 3).

### Evaluation of acetate formation and growth rates of seven *E. coli* strains

**Fig. 4:**
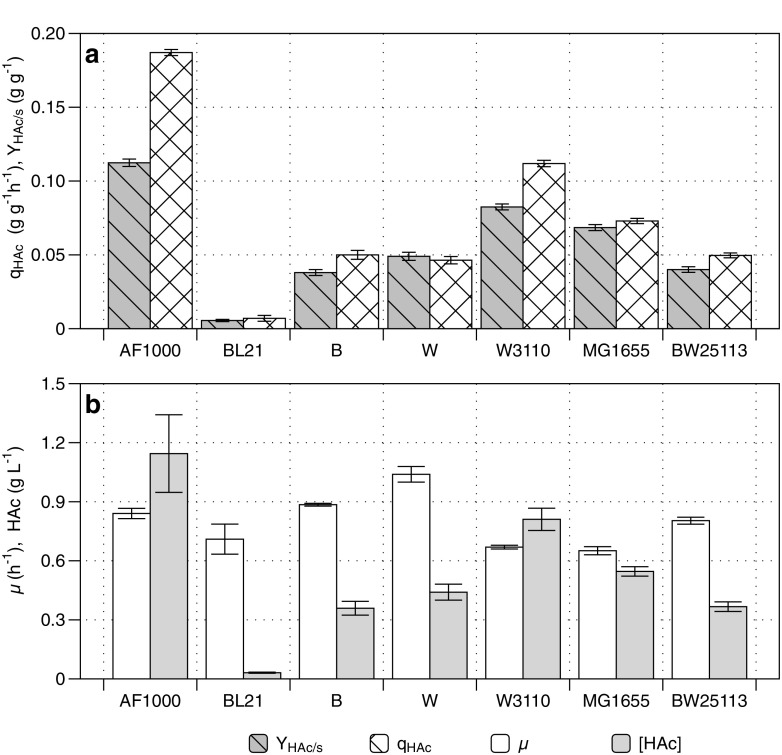
Acetate production and specific growth rate of *E. coli* B, BL21, W, W3110, AF1000, MG1655, and BW25113. Experiments were performed in batch bioreactor cultivations on minimal salt medium with glucose as carbon source. Box (a) shows the yield of acetate on glucose (Y_HAc/s_) and the specific acetate production rate (q_HAc_) during exponential growth. Box (b) shows the specific growth rate (μ) and the final acetate concentration (HAc) after 6 h. Bars represent the average and mean deviation of duplicate cultivations

To investigate if *E. coli* strains that inherently produce less acetate than AF1000 strain are more suitable as 3HB production platform, six additional strain backgrounds were first assessed for growth and acetate formation in the absence of a 3HB production pathway. This study investigated six promising *E. coli* strains, including two strains from group B (B and BL21) that are known to produce low amounts of acetate (Daegelen et al. 2009; Rosano and Ceccarelli 2014), one W strain known for its high growth rate in minimal medium and low acetate formation (Archer et al. 2011), and three additional K-12 strains (MG1655, W3110, BW25113) as well as the K-12 MC4100 derived AF1000 strain previously used (Baba et al. 2006; Blattner et al. 1997; Grenier et al. 2014; Hayashi et al. 2006; Sanden et al. 2003). The initial screening was performed during 6 h of exponential growth in bioreactor batch cultivations, which gives the high glucose concentrations required to assess overflow metabolism and acetate formation (Fig. 4). During these aerobic batch cultivations, acetate and biomass were the predominant products, and no significant amounts of other by-products were observed in the cultivation broth. *E. coli* BL21 produced the lowest concentration of acetate (0.03 g L^−1^) and its yield of acetate per glucose consumed (Y_HAc/s_) was 20 times lower than the Y_HAc/s_ of AF1000 (Fig. 4a). The strains W3110, MG1655, and AF1000 produced high amounts of acetate, while AF1000 produced the most (1.15 g L^−1^) (Fig. 4b). Important to highlight is that not all the K-12 strains produced high acetate concentrations. BW25113 produced the same amount of acetate as *E. coli* B, which was 3.2 times lower than the acetate concentration of AF1000. *E. coli* W produced a little more acetate than BW25113 and B strains, but this was solely caused by its very high growth rate (1.04 h^−1^) on minimal medium and ensuing increased sugar consumption and higher achieved CDW in the 6 h of the screening, as also exemplified by the equal q_HAc_ of these three strains. The growth rate of *E. coli* B was the second highest. BL21, MG1655, and W3110 had the lowest growth rates, which were similar at around 0.66 h^−1^ (Fig. 4b).

### Evaluation of *E. coli* strains for (*R*)-3-hydroxybutyrate production

**Fig. 5:**
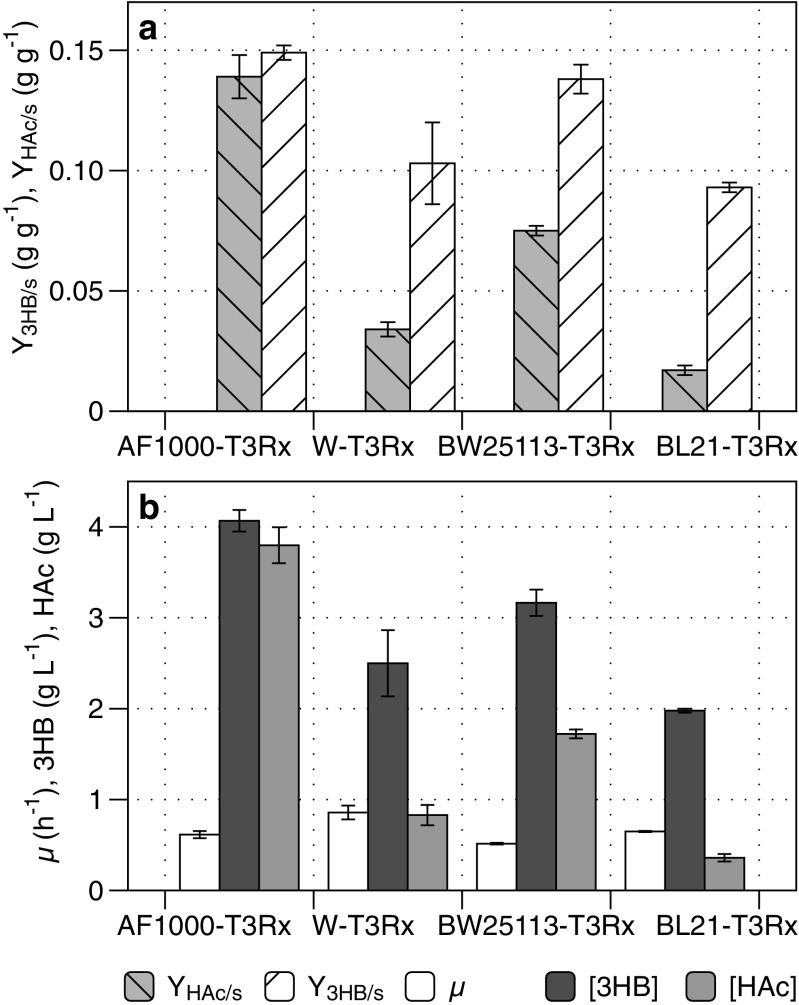
(*R*)-3-hydroxybutyrate production by four selected *E. coli* strains. Experiments were performed in batch bioreactor cultivations designed with two phases, an exponential growth phase and a nitrogen-depleted phase. To allow 3HB production, plasmid pJBGT3RX was inserted in BL21, W, AF1000, and BW25113, resulting in strain BL21-T3Rx, etc. Box (a) shows the yield of acetate on glucose (Y_HAc/s_) and the yield of (*R*)-3-hydroxybutyrate on glucose (Y_3HB/s_). Box (b) shows the specific growth rate (μ), and the final concentrations of (*R*)-3-hydroxybutyrate ([3HB]) and acetate ([HAc]) after 24 h. Bars represent the average and mean deviation of duplicate cultivations, with the exception of W-T3Rx, which was performed in triplicate

Based on the outcome of the preliminary screening (Fig. 4), strains BL21 (lowest acetate), W (high growth rate and lower acetate than AF1000), and BW25113 (K-12 strain with lower acetate than AF1000) were transformed with pJBGT3RX to assess their potential for 3HB production. The strains were evaluated in two-phase batch experiments with a total duration of 24 h, where the first phase was an exponential growth phase and the second phase was a nitrogen-depleted phase (Fig. 5). The duration of the exponential growth phase varied with the measured specific growth rate of each strain (Fig. 5b), ranging from 4.5 h for the fastest growing W-T3Rx strain (0.86 h^−1^) to 7.5 h for strain BW25113-T3Rx, which had a specific growth rate of 0.52 h^−1^ (Fig. 5b). While the growth rate differed, the maximum cell concentrations were similar for all the strains, which indicated a similar biomass yield on nitrogen. In the nitrogen-depleted phase, the CDW remained constant, while 3HB and acetate accumulated. In this low-cell-density screening, AF1000-T3Rx had both the highest 3HB and acetate yields and titers, with final concentrations of 4.07 g L^−1^ and 3.80 g L^−1^, respectively (Fig. 5b). The strains BW25113-T3Rx and W-T3Rx yielded intermediate results, with neither the highest 3HB titer nor the lowest acetate formation. Strain BL21-T3Rx showed by far the lowest acetate concentration (0.36 g L^−1^), but also had the lowest final 3HB concentration at 1.98 g L^−1^ (Fig. 5b). To avoid acetate accumulation and inhibition during increased cell density 3HB processes aiming for high product titers, a high ratio of 3HB over acetate formation would be highly beneficial. In that light, the ratios of the 3HB yield over the acetate yield were, respectively, 1.1 g g^−1^ for AF1000-T3Rx, 3 g g^−1^ for W-T3Rx, 1.8 g g^−1^ for BW25113-T3Rx, and 5.5 g g^−1^ for BL21-T3Rx.

### Evaluation of BL21-T3Rxzwf for 3HB production in high-cell-density nitrogen-limited fed-batch cultivation

**Fig. 6:**
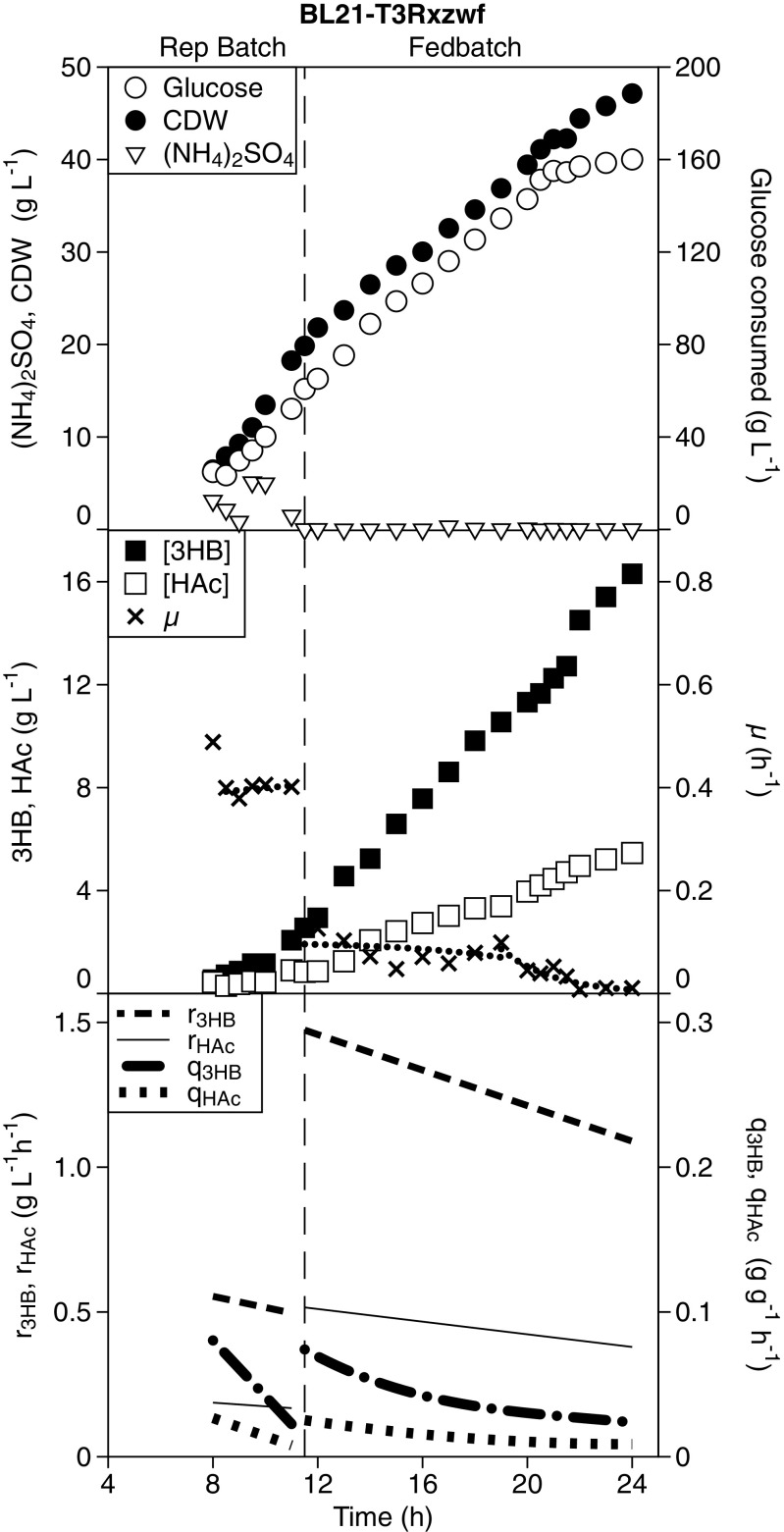
Nitrogen-limited fed-batch cultivation to evaluate (*R*)-3-hydroxybutyrate and acetate formation in the BL21 strain background. Bioreactor experiments were performed with a repeated batch phase followed by a constant feed nitrogen-limited fed-batch phase. The vertical dashed line marks the shift between repeated batch and nitrogen-limited fed batch. Samples were taken from OD_600_ = 10. BL21 was transformed with both plasmid, pJBGT3RX and pBADzwf. Symbols refer to cell dry weight (CDW, filled circles), accumulative glucose consumed (Glucose, open circles), (NH_4_)_2_SO_4_ concentration ((NH_4_)_2_SO_4_, inverted open triangles), specific growth rate (μ, crosses and dotted line), (*R*)-3-hydroxybutyrate concentration ([3HB], closed squares), acetate concentration ([HAc], open squares). The specific 3HB production rate (q_3HB_, dash-dotted line), volumetric 3HB productivity (r_3HB_, dashed line), specific acetate production rate (q_HAc_, dotted line) and volumetric acetate productivity (r_HAc_, solid line) as calculated from spline-fit of the raw data. Experiment was performed in duplicate; one representative experiment is shown in this figure and the duplicate is shown in Fig. S2 (online resource). The relevant AF1000-based control experiments can be found in Fig. 3a and Fig. S1a

Strain BL21 was selected for further evaluation at high cell-density in view of its beneficial 3HB over acetate ratio. After transformation with both plasmids pJBGT3RX and pBADzwf, the resulting BL21-T3Rxzwf strain was grown in repeated batch cultivation followed by a nitrogen-limited fed-batch phase (Fig. 6), thereby allowing quantitative comparison with the AF1000-based data obtained in an identical process (Fig. 3a). The 11.5-h duration of the repeated batch phase for BL21-T3Rxzwf was much shorter than the 19.5 h observed for AF1000-T3Rxzwf (Figs. 3a and 6), which is a direct consequence of the decreased acetate production by BL21-T3Rxzwf. In contrast to AF1000-T3Rxzwf (Fig. 3a), there was no growth inhibition for BL21-T3Rxzwf during the repeated batch phase, its growth rate remained constant and in spite of the added plasmid burden was 0.43 h^−1^ (Fig. 6). Both the biomass specific acetate production rate (q_HAc_) and volumetric acetate productivity (r_HAc_) were much lower for BL21 compared to AF1000 during the entire cultivation (Figs. 3a and 6). Pyruvate or other by-products formation were not detected neither in AF1000-T3Rxzwf nor in BL21-T3Rxzwf.

When the nitrogen-limited fed-batch phase started, the acetate concentration was 0.86 g L^−1^ (Fig. 6), which it was almost 12 times lower than AF1000-T3Rxzwf (Fig. 3a). The decreased acetate inhibition for BL21-T3Rxzwf, resulted in increased metabolic activity, as shown by a 3-fold higher biomass-specific 3HB production rate at the start of the nitrogen-limited feed, which translated to a highest observed volumetric productivity of 1.52 g L^−1^ h^−1^ (Fig. 6). During the nitrogen-limited fed batch, acetate formation by BL21-T3Rxzwf increased compared to the repeated batch phase, but the final concentration of 5.5 g L^−1^ (Fig. 6) remained well below the final acetate concentration of 12.1 g L^−1^ obtained with the AF1000 strain (Fig. 3a). Consequently, growth and 3HB production by the BL21-T3Rxzwf continued much longer into the nitrogen-limited fed batch than was observed for AF1000-T3Rxzwf (Figs. 3a and 6), resulting in a 2.3-fold increase in the final 3HB titer (16.31 g L^−1^ versus 7.04 g L^−1^), a 2.3-fold higher final CDW (47.14 g L^−1^ versus 20.10 g L^−1^) and a 3-fold higher volumetric 3HB productivity during the nitrogen-limited fed-batch phase (1.27 g L^−1^ h^−1^ versus 0.42 g L^−1^ h^−1^). Deletion of *pta* and/or *poxB* in the BL21 strain background did not improve 3HB formation compared to BL21-T3Rxzwf in nitrogen-depleted batch nor nitrogen-limited fed batch (Fig. S3, Fig. S4, online resource).

## Discussion

To control acetate formation in high-cell-density cultivations without compromising cellular performance is not a trivial task, especially for product formation pathways using acetyl-CoA as an essential precursor, and here further also requiring a thioesterase as the final step in the pathway, which hitherto uses both acetyl-CoA and 3HB-CoA as substrates. In this study, use of the BL21 strain background for 3HB production was more beneficial than targeted metabolic engineering of the acetyl-CoA branch point through deletion of *iclR*, *pta*, and/or *poxB*. Interestingly, screening solely for 3HB titers in simple low-density batch cultivations would not have identified the potential of this strain, reaffirming the importance to screen with the final process conditions in mind (Crater and Lievense 2018; Noorman and Heijnen 2017). The most suitable strain for 3HB production resulted to be BL21 because of its low acetate production and its favorable specific growth rate in minimal medium in spite of the added metabolic load of the producing genes. Despite the close relation between *E. coli* B and BL21 (Daegelen et al. 2009), this study showed large differences in acetate formation, with *E. coli* B (ATCC #11303) producing amounts more similar to the K-12 derived BW25113 instead of BL21. Several studies on acetate formation stated that *E. coli* BL21 produces low acetate titers during high glucose cultivations because this strain has a more active glyoxylate shunt pathway (Phue et al. 2005; van de Walle and Shiloach 1998; Waegeman et al. 2011). The glyoxylate operon is negatively regulated by *iclR* and, according to (Waegeman et al. 2011), deletion of *iclR* increased the flux through the TCA cycle and reduced acetate formation by increasing the biomass production in MG1655. However, in this study, *iclR* deletion did not decrease acetate formation in AF1000 (Fig. 2), further illustrating the importance of evaluating the strain dependent impact of engineering strategies and process conditions.

Although deletion of *pta* and/or *poxB* was to some extent successful in decreasing acetate formation, this did not result in increased 3HB production in high-cell-density fed-batch cultivations. The enzyme Pta is considered the predominant responsible for acetate formation in *E. coli* during aerobic exponential growth (De Mey et al. 2007; Dittrich et al. 2005), as was also confirmed in this study (Figs. 2 and 3). In contrast, PoxB is mostly associated with acetate formation in the stationary phase (Dittrich et al. 2005), and low expression levels of *poxB* have previously been hypothesized as the underlying cause for the lower acetate formation in BL21 (Phue et al. 2005). However, no positive impact of *poxB* deletion was observed in batch cultivation during either the exponential growth phase or the nitrogen-depleted phase in the AF1000 background used in this study (Fig. 2d). When AF1000Δ*pta* and AF1000Δ*pta*Δ*poxB* were tested in high-cell-density cultivations, the specific growth rates decreased continuously in the repeated batch phase, even though acetate concentrations were not too high (Fig. 3). However, deletion of *pta* and/or *poxB* resulted in dramatically increased pyruvate formation. The decreased acetate formation in combination with a limited capacity of the 3HB production pathway might have resulted in increased levels of acetyl-CoA, which in turn can decrease the activity of the pyruvate dehydrogenase complex due to an allosteric inhibition of the transacetylase component (E_2_) (Berg et al. 2002; Chang et al. 1999; Sanwal 1970). In line with this, pyruvate excretion was particularly high under nitrogen-depleted or nitrogen-limited conditions, where the biosynthetic demand for pyruvate and ATP is decreased, thereby also decreasing the flux through the TCA cycle, potentially decreasing the demand for acetyl-CoA. The total sum of acetate and pyruvate diverted away from 3HB production was, respectively, 169, 78, and 76 mM acetyl-CoA-equivalents in AF1000-T3Rxzwf and the corresponding Δ*pta* and Δ*pta*Δ*poxB* strains at the end of the batch (Fig. 3). Despite this, the specific 3HB production rates were lower in the Δ*pta* and Δ*pta*Δ*poxB* strain compared to the control. In combination with the lower pKa and oleyl-water partitioning coefficient of pyruvate compared to acetate (Collander 1951; Dawson 1959), the impact of pyruvate formation on 3HB production and growth in high-cell-density cultivations seems to be larger than solely expected based on competition for carbon or the weak-organic-acid toxicity. The observed decreased product formation as well as decreased growth rates (Fig. 3) might be exacerbated by inhibition of the phosphotransferase system (PTS) through increased pyruvate levels (Deutscher et al. 2006).

The limited impact of the abovementioned deletions alludes to limitations in the 3HB production pathway. The 3HB formation pathway used in this, and many other studies, relies on native *E. coli* acyl-CoA thioesterases, such as *fadM*, *tesA*, *tesB*, *ybgC*, *ydiI*, and *yciA*, which are also active with acetyl-CoA as a substrate (Clomburg et al. 2012). To decrease competition for acetyl-CoA of the 3HB production pathway and acetate formation, it is crucial to engineer thioesterases with a very high specificity for 3HB-CoA (McMahon and Prather 2014), or acyl-CoAs depending on the desired product, compared to acetyl-CoA. Additionally, to maximally benefit from the strong thermodynamic pull of the thioesterase reaction in (indirectly) decreasing acetyl-CoA levels and increasing 3HB formation, it is important to ensure that the activities of thiolase and reductase are not limiting the pull of the product formation pathway. As an example, acetoacetyl-CoA reductases with a preference or specificity for NADPH, in combination with increased supply of NADPH (Perez-Zabaleta et al. 2016) were beneficial for 3HB production. The reductase (*rx*) from *H. boliviensis*, for instance, has a 16 times higher affinity for acetoacetyl-CoA (k_cat_/K_m_ = 11.1 s^−1^ μM^−1^) than the reductase from the well-known PHB producer *C. necator* (k_cat_/K_m_ = 0.685 s^−1^ μM^−1^) (Matsumoto et al. 2013; Perez-Zabaleta et al. 2016). In addition to providing leads for “pathway oriented” approaches, this study demonstrated how careful selection of the strain background together with evaluation of all cultivation parameters, such as specific growth rate and by-product formation, can result in high volumetric productivities of 3HB (r_3HB_ = 1.52 g L^−1^ h^−1^) and high 3HB titers (16.3 g L^−1^). Improvements in productivity and titers as a result of reduced acetate toxicity and improved growth can also be used to improve high-cell-density cultivations for other products and pathways.

## Electronic supplementary material

ESM 1 (PDF 337 kb)
