# Supplementary material for: Comparison of engineered Escherichia coli AF1000 and BL21 strains for (R)-3-hydroxybutyrate production in fed-batch cultivation
Source: Appl Microbiol Biotechnol. 2019 May 18;103(14):5627–39. doi: 10.1007/s00253-019-09876-y (PMC6597613; doi:10.1007/s00253-019-09876-y)
Supplement: Supplementary file 1 — (PDF 337 kb) [file 253_2019_9876_MOESM1_ESM.pdf]

Comparison of engineered *Escherichia coli* AF1000 and BL21 strains for *(R)*-3-hydroxybutyrate production in fed-batch cultivation

Applied Microbiology and Biotechnology

Authors: Mariel Perez-Zabaleta[1,2], Mónica Guevara-Martínez[1,2], Martin Gustavsson[1], Jorge Quillaguamán[2], Gen Larsson[1] and Antonius J. A. van Maris[1]

Affiliations:

1 School of Engineering Sciences in Chemistry, Biotechnology, and Health (CBH), Department of Industrial Biotechnology, KTH Royal Institute of Technology, Stockholm, Sweden.

2 Center of Biotechnology, Faculty of Science and Technology, Universidad Mayor de San Simón, Cochabamba, Bolivia.

____

*Corresponding author: Antonius J. A. van Maris, tonvm@kth.se

Table S1 List of primers used for gene deletions and PCR verification

Gene Knockout	Plasmid used	Primer name	Primer sequence 5'-->3'
pta	pKD3	H1(pta)P1	GCTGTTTTGTAACCCGCCAAATCGGCGGTAACGAAAGAGGATAAACCGTGTGTGTAGGCTGGAGCTGCTTC
		H2(pta)P2	AGCGCAAAGCTGCGGATGATGACGAGATTACTGCTGCTGTGCAGACTGGCCATGGTCCATATGAATATCCTCC
		Sequence_fw_pta	TGATCATGAACGCAACCTGG
		Confirm_rev_CM	CCCAATGGCATCGTAAAGAACA
		Sequence_rev_pta	GCAATTCATTGATGCAGCGC
		Sequence_fw_pta	TGATCATGAACGCAACCTGG
poxB	pKD4	H1(poxB)P1	GATGAACTAAACTTGTTACCGTTATCACATTCAGGAGATGGAGAACCATGTGTGTAGGCTGGAGCTGCTTC
		H2(poxB)P2	CCTTATTATGACGGGAAATGCCACCCTTTTTACCTTAGCCAGTTTGTTTTGCCATGGTCCATATGAATATCCTCC
		Sequence_fw_poxB	CCGGTGAATATACGGTGAGC
		Confirm_rev_Kan	CCACAGTCGATGAATCCAGA
		Sequence_rev_poxB	TCGCAGTGACTGAGCAGAGC
		Sequence_fw_poxB	CCGGTGAATATACGGTGAGC
iclR	pKD3	H1P1_forward	GAGACTGTCATGGTCGCACCCATTCCCGCGAAACGCGGCAGAAAACCCGCGTGTAGGCTGGAGCTGCTTC
		H2P2_reverse	GCCCGCCAGAAAAAGTCAGCGCATTCCACCGTACGCCAGCGTCACTTCCTGGTCCATATGAATATCCTCCTTAGTTCC
		Confirm_ fw	GAGACTGTCATGGTCGCACCC
		Confirm_rev	CCCAATGGCATCGTAAAGAACA
		Sequencing_fw	TACTGGCATAAACGCATCTG
		Sequencing_rev	CACTCAGGTATGATGGGCAG

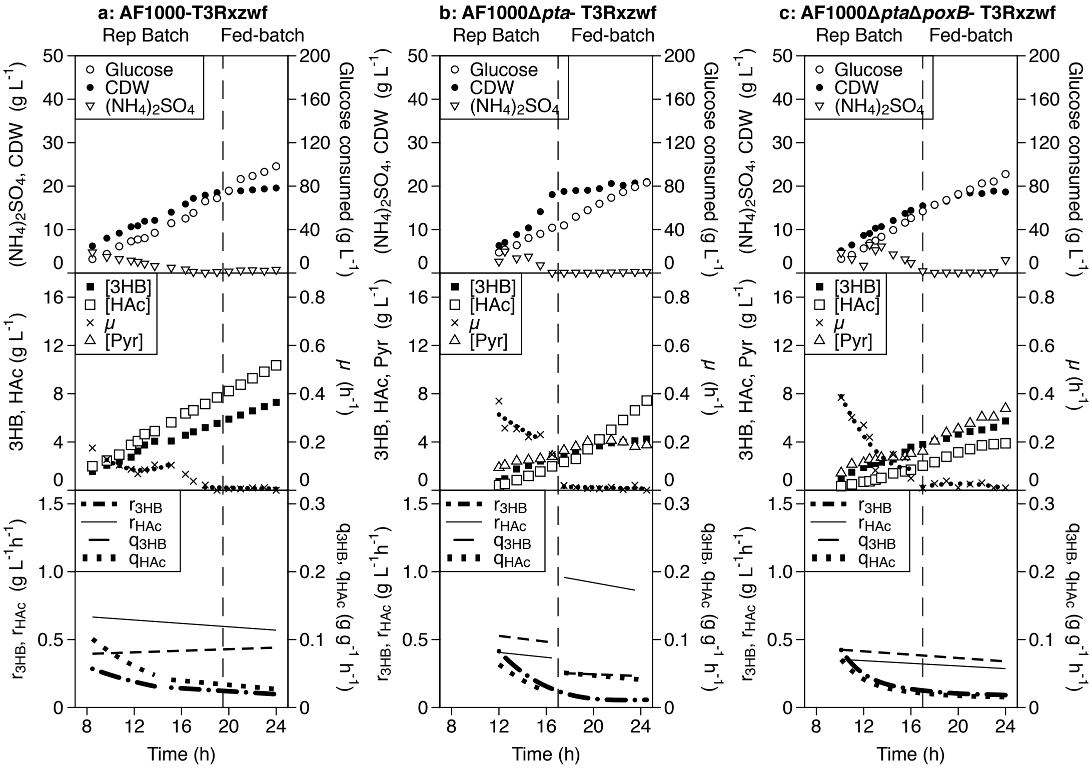

Fig. S1 Duplicate experiments, nitrogen-limited fed-batch cultivations to evaluate (*R*)-3-hydroxybutyrate and acetate formation by (a) AF1000 (b) AF1000Δ*pta* and (c) AF1000Δ*pta*Δ*poxB*. Experiments were performed in fed-batch bioreactor cultivations with constant feed and were designed with a repeated batch phase followed by a nitrogen-limited phase. The vertical dashed line marks the shift between repeated batch and nitrogen-limited fed batch. Samples were taken from OD_{600}=10. The strains were transformed with both plasmids, pJBGT3Rx and pBADzwf. Symbols refer to cell dry weight (CDW, filled circles), accumulative glucose consumed (Glucose, open circles), $(NH_4)_2SO_4$ concentration ($(NH_4)_2SO_4$, inverted open triangles), specific growth rate (μ, crosses and dotted line), (*R*)-3-hydroxybutyrate concentration ([3HB], closed squares), acetate concentration ([HAc], open squares), pyruvate concentration ([Pyr], open triangles). The specific 3HB production

rate (q_{3HB}, dash-dotted line), volumetric 3HB productivity (r_{3HB}, dashed line), specific acetate production rate (q_{HAc}, dotted line) and volumetric acetate productivity (r_{HAc}, solid line) as calculated from spline-fit of the raw data

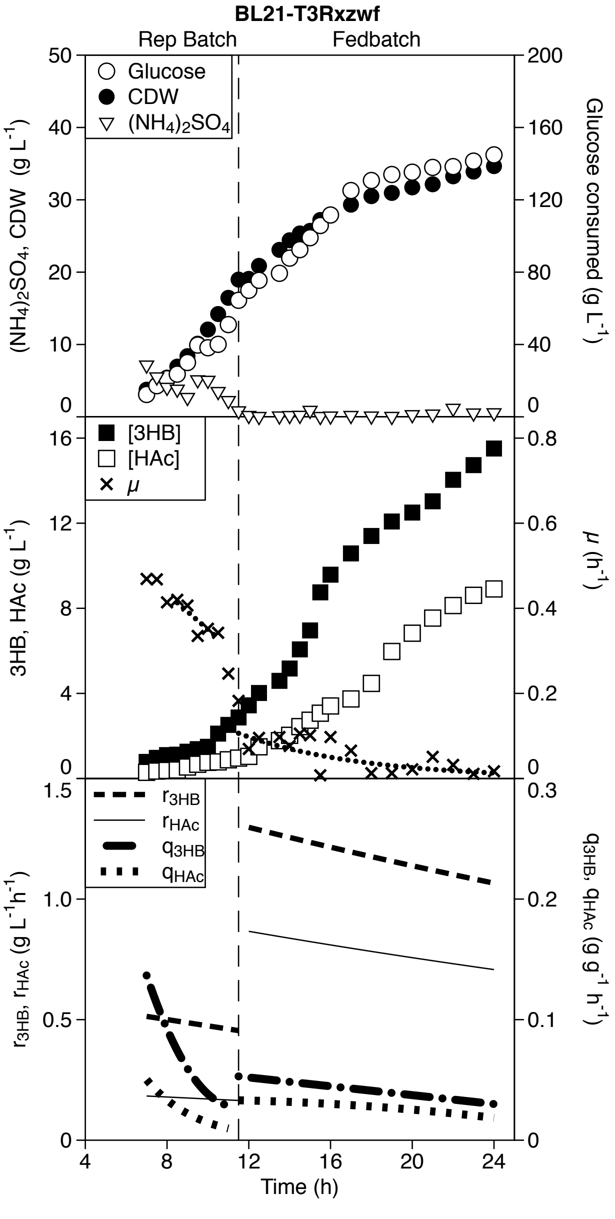

Fig. S2 Duplicate experiment, nitrogen-limited fed-batch cultivation to evaluate (*R*)-3-hydroxybutyrate and acetate formation in the BL21 strain background. Bioreactor experiments were performed with a repeated batch phase followed by a constant feed nitrogen-limited fed-batch phase. The vertical dashed line marks the shift between repeated

batch and nitrogen-limited fed batch. Samples were taken from OD_{600}=10. BL21 was transformed with both plasmid, pJBGT3Rx and pBADzwf. Symbols refer to cell dry weight (CDW, filled circles), accumulative glucose consumed (Glucose, open circles), $(NH_4)_2SO_4$ concentration ($(NH_4)_2SO_4$, inverted open triangles), specific growth rate (μ, crosses and dotted line), (*R*)-3-hydroxybutyrate concentration ([3HB], closed squares), acetate concentration ([HAc], open squares). The specific 3HB production rate (q_{3HB}, dash-dotted line), volumetric 3HB productivity (r_{3HB}, dashed line), specific acetate production rate (q_{HAc}, dotted line) and volumetric acetate productivity (r_{HAc}, solid line) as calculated from spline-fit of the raw data. Experiment was performed in duplicate

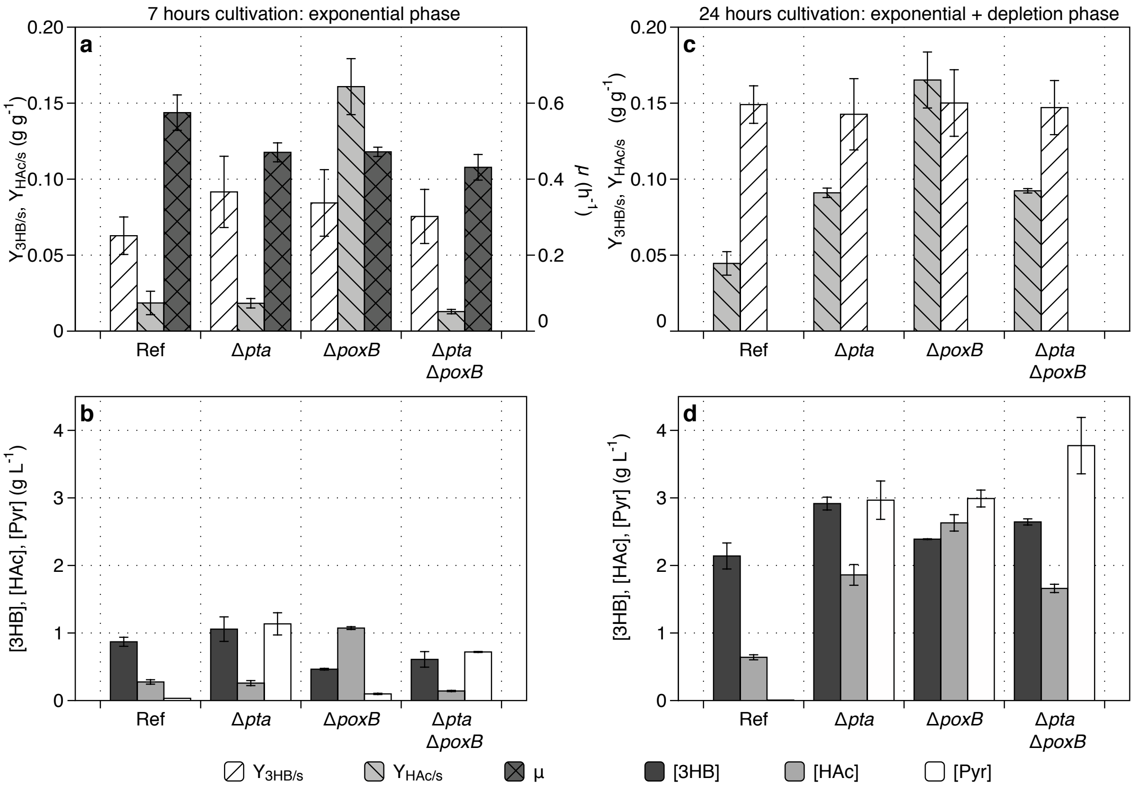

Fig. S3 Evaluation of (*R*)-3-hydroxybutyrate and acetate formation of *pta* and/or *poxB* deletions in the BL21 strain background. Experiments were performed in batch bioreactor cultivations designed with two phases, exponential growth phase and nitrogen-depleted phase. Both plasmids, pJBGT3Rx and pBADzwf, were inserted in the BL21 reference and the knock out strains. Boxes (a) and (b) show cultivations parameters during the exponential growth phase. Boxes (c) and (d) show the parameters after 24 hours of cultivation. Boxes (a) and (c) show the yield of acetate on glucose ($Y_{HAc/s}$) and the yield of (*R*)-3-hydroxybutyrate on glucose ($Y_{3HB/s}$). Additionally, box (a) shows the specific growth rate (μ). Boxes (b) and (d) show the concentrations of (*R*)-3-hydroxybutyrate ([3HB]), acetate ([HAc]) and pyruvate ([Pyr]). Bars represent the average and mean deviation of duplicate cultivations

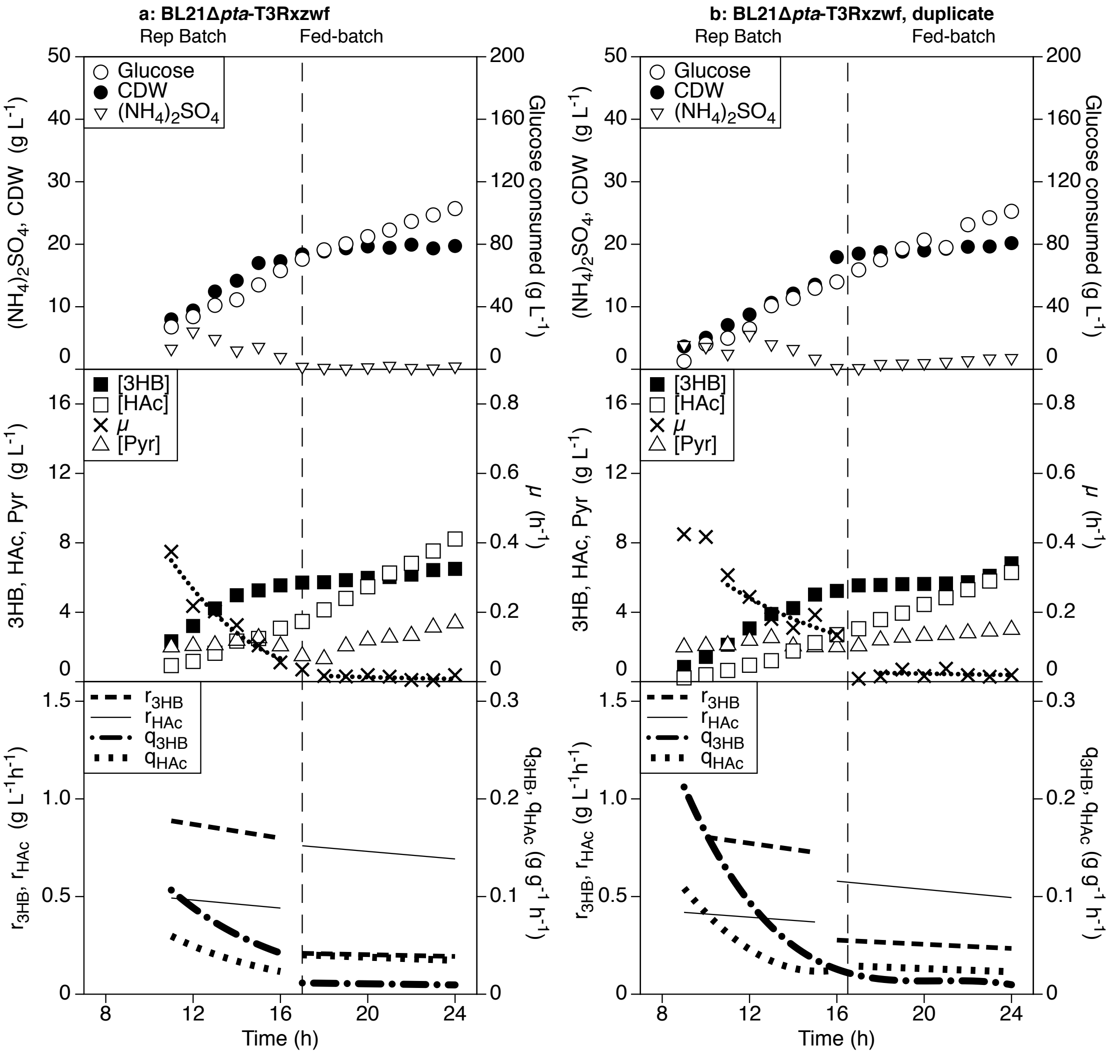

Fig. S4 Nitrogen-limited fed-batch cultivations to evaluate (*R*)-3-hydroxybutyrate and acetate formation by BL21Δ*pta*. Cultivations (a) and (b) are duplicate experiments. Experiments were performed in fed-batch bioreactor cultivations with constant feed and were designed with a repeated batch phase followed by a nitrogen-limited phase. The vertical dashed line marks the shift between repeated batch and nitrogen-limited fed batch. Samples were taken from OD_{600}=10. The strain was transformed with both plasmid, pJBGT3Rx and pBADzwf. Symbols refer to cell dry weight (CDW, filled circles), accumulative glucose consumed (Glucose, open circles), $(NH_4)_2SO_4$ concentration ($(NH_4)_2SO_4$, inverted open triangles), specific growth rate (μ, crosses and dotted line), (*R*)-

3-hydroxybutyrate concentration ([3HB], closed squares), acetate concentration ([HAc], open squares), pyruvate concentration ([Pyr], open triangles). The specific 3HB production rate (q_{3HB}, dash-dotted line), volumetric 3HB productivity (r_{3HB}, dashed line), specific acetate production rate (q_{HAc}, dotted line) and volumetric acetate productivity (r_{HAc}, solid line) as calculated from spline-fit of the raw data
